# Pachychoroid Pigment Epitheliopathy and Choroidal Thickness Changes in Coeliac Disease

**DOI:** 10.1155/2019/6924191

**Published:** 2019-02-13

**Authors:** Selim Bolukbasi, Burak Erden, Akin Cakir, Alper Halil Bayat, Mustafa Nuri Elcioglu, Serap Yurttaser Ocak, Yasemin Gokden, Mine Adas, Zekiye Nur Asik

**Affiliations:** ^1^University of Health Sciences, Okmeydani Training and Research Hospital, Department of Ophthalmology, Istanbul, Turkey; ^2^University of Health Sciences, Okmeydani Training and Research Hospital, Department of Gastroenterology, Istanbul, Turkey

## Abstract

**Purpose:**

To evaluate choroidal thickness in patients with coeliac disease (CD) using spectral domain optical coherence tomography (SD-OCT) and to compare the results to normal eyes.

**Methods:**

Seventy patients with CD and 70 healthy controls were included in this prospective, comparative study. All participants underwent a complete ophthalmologic evaluation and SD-OCT. Subfoveal, nasal (nasal distance to fovea 500 *μ*m, 1000 *μ*m, and 1500 *μ*m), and temporal (temporal distance to fovea 500 *μ*m, 1000 *μ*m, and 1500 *μ*m) choroidal thickness measurements were performed using SD-OCT.

**Results:**

There were no significant differences in sex, ages, and axial lengths between the groups (*p*=1.0, *p*=0.601, *p*=0.314, respectively). The mean choroidal thickness measurements at all predefined measurement point areas were higher in the coeliac group than in the healthy controls (*p* < 0.001). Of all patients with coeliac disease (70 eyes of 70 patients), 64 eyes (84.2%) had uncomplicated pachychoroid (UCP), one eye had pachychoroid pigment epitheliopathy (PPE), and five eyes in the UCP group had PPE in fellow eyes.

**Conclusion:**

It is probable that systemic inflammation in coeliac patients causes the enlargement of choroidal vessels and increasing choroidal thickness. PPE, which is believed to be the precursor of central serous chorioretinopathy, can be observed in coeliac patients.

## 1. Introduction

Coeliac disease (CD) is a multifactorial, chronic, and polygenic disorder of the small bowel triggered by an immune response to the gluten proteins of wheat, barley, and rye [1]. The main pathology of the disorder is in the small proximal intestine, and untreated CD may cause the loss of intestinal villi. As a result, patients have malabsorption findings, such as iron, vitamin B12 deficiency, and anaemia. CD increases patient susceptibility to some autoimmune diseases and cancers, such as thyroiditis and lymphoma [2].

The prevalence of CD is estimated to be approximately 1% in most Western countries, although many individuals with CD remain undiagnosed. CD individuals have a susceptibility to other autoimmune diseases, such as diabetes mellitus type 1 and thyroiditis. HLA-DQ2 and HLA-DQ8 (human leukocyte antigen) are found in 95% of the patients [3].

Gluten is degraded by gastrointestinal tract enzymes into peptides. In susceptible individuals, these peptides are presented to T lymphocytes after binding HLA-DQ2 or HLA-DQ8 heterodimers expressed on the surface of antigen-presenting cells in the intestinal lamina propria [4, 5]. Gluten-activated T-cells induce proinflammatory cytokines (mainly interferon-gamma (IFN-*γ*), interleukin- (IL-) 21, and IL-17), which are responsible for a cytotoxic effect on the epithelium [5, 6].

Some studies have shown a relationship between CD and ocular surface diseases, like dry eye. Dendritic cells and natural killer B and T cells are responsible for ocular surface inflammation [7, 8]. Besides the autoimmune response, deficiencies of vitamins due to malabsorption might contribute to dry eye in CD patients.

In the literature, no study has specifically focussed on the posterior segment of the eye of the patient with CD. This study aimed at determining posterior segment changes, including retina and choroid, using spectral domain optical coherence tomography (SD-OCT).

## 2. Materials and Methods

This prospective, comparative clinical study was carried out between December 2017 and March 2018 in Okmeydani Training and Research Hospital, Istanbul, Turkey. Informed consent was obtained from all participants, and the study was carried out in agreement with the Declaration of Helsinki for research involving human subjects. Two subject groups were considered in this study. One consisted of patients with CD who were being followed at the gastroenterology clinic, while the other, the control group, was formed by age- and gender-matched, healthy subjects examined at the eye clinic of the same hospital. The diagnosis of CD was performed by the gastroenterology department according to the ESPGHAN criteria [9]. The patients participating in this study had no vitamin deficiencies and were following a gluten-free diet.

Systolic and diastolic blood pressures were measured. The blood pressure values of coeliac patients and control groups were within the normal range (systolic < 120 mmHg; diastolic < 80 mmHg). In the coeliac group, blood tests measuring vitamin D, vitamin B12, haemoglobin, iron, and thyroid functional tests (T3, T4, and TSH) were in the normal range.

Exclusion criteria included any retinal pathologies (such as diabetic retinopathy, epiretinal membrane, or vitreomacular traction syndrome); ocular surgery; previous ocular trauma; uveitis; congenital malformations of the eye; significant media opacities precluding fundus examination and/or imaging; abnormal thyroid functionality tests; inflammatory disorders, such as ankylosing spondylitis, Behcet disease, and Familial Mediterranean fever (FMF); best-corrected visual acuity below 20/20; ocular hypertension or glaucoma; systemic arterial hypertension; pregnancy; neurodegenerative disorders; spherical equivalent > ±3.0 dpt; a history of smoking or alcohol intake; or using any medication, such as oral steroids, within the last three months.

**Figure 1:**
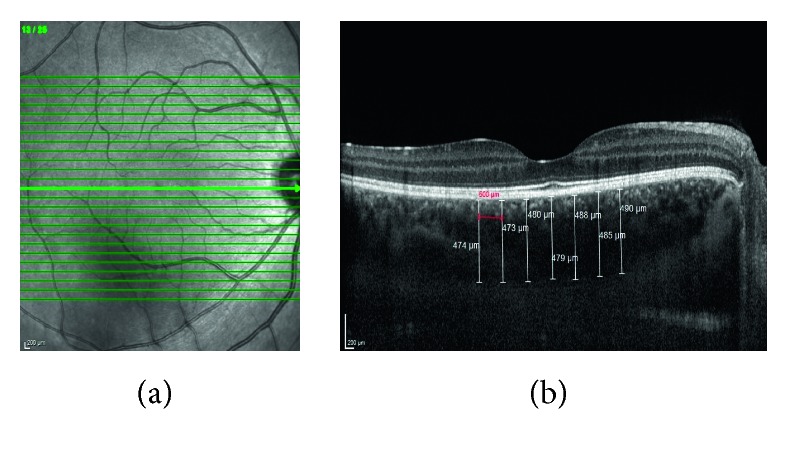
A representative enhanced depth imaging (EDI) mode of spectral domain optical coherence tomography (SD-OCT) image showing the choroidal thickness measurements of a coeliac patient.

All participants underwent a complete ophthalmic evaluation, including slit-lamp biomicroscopy, dilated fundus examination, B-scan ultrasonography, Goldmann applanation tonometry, and Snellen visual acuity testing. Axial length (AL) was measured with AL-Scan optical biometer (Nidek Co., Gamagori, Japan). The enhanced depth imaging (EDI) mode of an SD-OCT (Spectralis HRA + OCT; Heidelberg Engineering Inc., Heidelberg, Germany) was used to evaluate choroidal thickness. Twenty-five sections composed of 40 averaged scans were obtained within a 10° × 20° rectangle centred on the fovea. The choroidal thickness was measured between the hyperreflective retinal pigment epithelium-Bruch's membrane complex and the hyperreflective scleral/choroidal junction (manually drawn by the examiner). All SD-OCT measurements were performed between 10:00 a.m. and 11:00 a.m. Choroidal thickness was measured and noted manually by two independent graders, and both graders determined their own measurement positions. The average measurements were used in statistical analysis. Only the right eye of each participant was evaluated for statistical analysis. The subfoveal, nasal, (nasal distance to fovea 500 *μ*m, 1000 *μ*m, and 1500 *μ*m) and temporal (temporal distance to fovea 500 *μ*m, 1000 *μ*m, and 1500 *μ*m) choroidal thickness measurements were performed manually (Figure 1).

Choroidal thicknesses of eyes with pachychoroid phenotype greater than 300 *μ*m together with and without retinal pigment epithelium abnormalities were evaluated as uncomplicated pachychoroid (UCP) and pachychoroid pigment epitheliopathy (PPE), respectively.

Statistical analyses were performed using the SPSS software, version 21. The variables were investigated using visual and analytical methods to determine normality. The Student's *t*-test and Mann–Whitney *U* test were used to compare these parameters between groups. The effects of age, gender, and axial length were adjusted using the ANCOVA test. The Spearman correlation coefficient was performed to evaluate the correlation between the duration of coeliac disease and choroidal thickness. A *p* value of less than 0.05 was statistically significant. Effective predictors of UCP were investigated using binary logistic regression analysis.

## 3. Results

**Figure 2:**
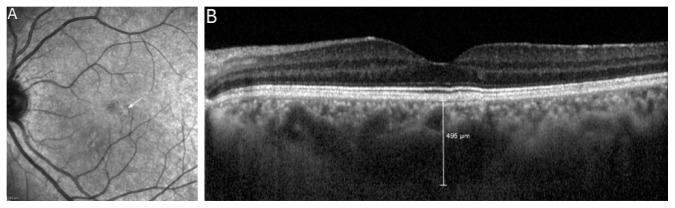
An infrared reflectance image (A) and enhanced depth imaging (EDI) mode of spectral domain optical coherence tomography (SD-OCT) image (B) of a 32-year-old female patient with coeliac disease. Slight hyperreflectivity appears on the infrared reflectance image in the parafoveal region (white arrow). In the SD-OCT image, pachyveins (enlargement of Haller's layer) under the retinal pigment epithelium change, and thick choroid (subfoveal choroidal thickness: 495 *μ*m) is seen.

**Figure 3:**
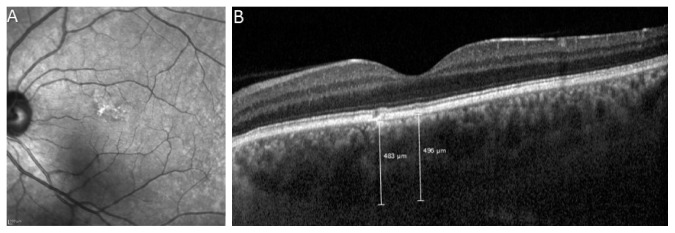
An infrared reflectance image (A) and enhanced depth imaging (EDI) mode of spectral domain optical coherence tomography (SD-OCT) image (B) of a 42-year-old male patient with coeliac disease. Irregular hyperreflectivity appears on the infrared reflectance image of the lesion. In the SD-OCT image, pachyveins (enlargement of Haller's layer) under the retinal pigment epithelium (RPE) change and thick choroid (subfoveal choroidal thickness: 496 *μ*m) is seen. Choroidal thickness is 483 *μ*m under the RPE changes.

Seventy eyes of 70 patients with CD were enrolled as the study group, and 70 eyes of 70 patients were enrolled as the control group in this prospective, case-control study.  Table 1 shows the demographics and ocular characteristics of the subjects. There were no significant differences in sex, age, and axial lengths between the groups (*p*=1.0, *p*=0.601, and *p*=0.314, respectively). The mean age was 37.4 ± 12.8 years (range: 13–65) in the coeliac group and 38.9 ± 11.2 years (range: 13–58) in the control group. 74.3% of the patients were female. The mean duration of coeliac disease was 4.6 ± 5.01 years (range: 1–26). The best-corrected visual acuity was 20/20 in both the groups. The mean intraocular pressure was 15.7 ± 2.3 mmHg in the coeliac group and 15.4 ± 2.2 mmHg in the control group. Of all patients with coeliac disease (70 eyes of 70 patients), 64 eyes (84.2%) had UCP, and one eye had PPE, and five eyes of the UCP group had PPE in fellow eyes. The SD-OCT and IR images of two patients with PPE are presented in Figures 2 and 3.

The UCP was significantly statistically correlated with coeliac disease. *p* < 0.001, Exp [B]:0.044, 0.016–0.120; 95% CI interval. Age and axial length were not found effective on UCP. (Age: *p*=0.116, Exp [B]:0.970, 0.933–1.008; 95% CI interval) (Axial length: *p*=0.979, Exp [B]:0.983, 0.277–3.487; 95% CI interval).

**Figure 4:**
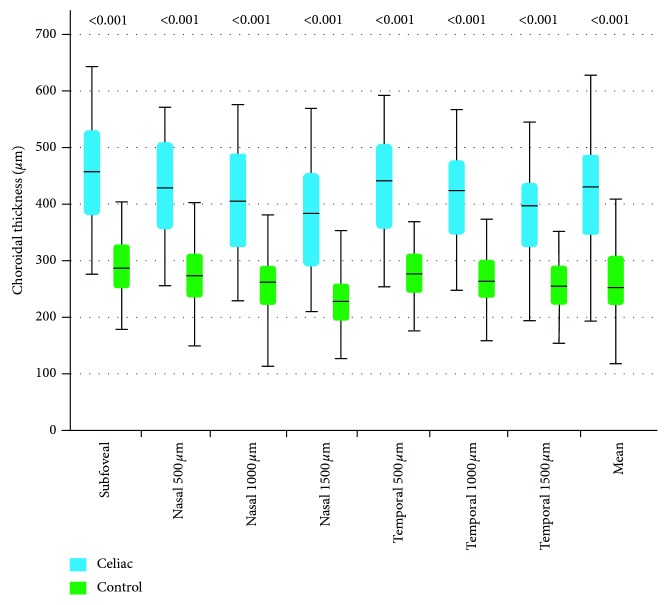
Choroidal thickness measurements. The values above the boxes show statistical significance when comparing the choroidal thickness measurements of the coeliac and control groups.

The mean choroidal thickness measurements at all subfoveal, nasal, and temporal points were higher in the coeliac group than in the control group. The results are shown in Table 2.  Figure 4 shows that the choroidal thickness was significantly thicker at all predefined measurement points in patients with CD (all *p* < 0.001).

The effects of age, gender, and axial length were adjusted by using the ANCOVA test, and the statistical significance was still remarkable regarding choroidal thickness between the groups. Age was also found to have a statistically significant effect on choroidal thicknesses.

When the coeliac group was assessed in isolation, a negative correlation between the duration of coeliac disease and the choroidal thicknesses at all measurement points was observed.  Table 3 shows the correlation coefficient results.

## 4. Discussion

The choroid plays an important role in the pathophysiology of numerous chorioretinal diseases, such as Vogt–Koyanagi–Harada and central serous chorioretinopathy [10, 11]. Detailed visualisation of the choroid and the measurement of choroidal thickness using EDI-OCT enable researchers to understand the pathologic processes within the choroid. In this study, we evaluated the choroidal thickness changes in CD and compared them with a normal population.

CD is a chronic, immune-mediated inflammatory disease. Typical findings of the disease include villous atrophy, crypt hyperplasia, and increased infiltration by intraepithelial lymphocytes in the small intestine [12]. In the literature, various inflammatory cytokines and antibodies were detected in the tissue and blood samples of coeliac patients. Immunological manifestations include IgA antibodies to gliadin; autoantigens tissue transglutaminase- (tTG-) 2 and endomysium; and proinflammatory cytokines interferon- (IFN-) *γ*, interleukin- (IL-) 17A, and IL-21 [13, 14]. Lahat et al. have shown increasing levels of IL-2, IFN-gamma, TNF-beta, IL-4, and IL-10 in the peripheral blood samples of coeliac patients when compared to a control group [15].

Few studies have examined the relationship between CD and ocular pathologies. One study indicated an increased risk of uveitis in the CD population [16]. In other case presentations, researchers demonstrated that scleritis and xerophthalmic fundus could be seen in the CD population [17, 18]. Uzel et al. reported that the impression cytology grading score was significantly higher in the CD group than in the control group resulting from ocular surface inflammation, and increasing levels of IL-1, IL-6, TNF-alpha, and IL-17 have been found in the cornea and conjunctiva epithelium [7].

The mean choroidal thickness measurements at all subfoveal, nasal, and temporal points were found to be higher in the coeliac group than in the control group of this study. Several studies have indicated that choroidal thickness increases as part of various inflammatory diseases. As a result of inflammation, cytokines increase the choroidal vascular permeability and choroidal thickness. Vogt–Koyanagi–Harada disease is one of the most studied diseases, showing that choroidal thickness increases secondary to inflammation. In these patients, increased choroidal thickness returns to normal thickness levels and resolves exudative retinal detachment after corticosteroid therapy [19]. Choroidal thickness in Behcet's disease during the acute phase is higher than in the remission phase, and following the regression of ocular inflammation with infliximab therapy, choroidal thickness decreases [20]. There are also reports of increased choroidal thickness in systemic inflammatory diseases, such as FMF disease and psoriasis, in the literature. In Gundogan et al.'s study, it was observed that the choroidal thickness increases in acute exacerbation periods of FMF disease [21]. Ersan et al. indicated that IL-8, IL-17, and TNF-*α* are related to the development of psoriasis, and they showed that, in severe psoriasis patients, the choroidal thickness was increased [22]. Increasing levels of cytokines in the peripheral blood samples [15], cornea, and conjunctiva epithelium [7] in CD suggest that the thickening choroid in CD may be associated with inflammation.

In our study, all SD-OCT measurements were performed at the same time of day to exclude diurnal variation [23]. To avoid interobserver variation, both independent graders measured the choroidal thickness at the same time.

This study found that there was a negative correlation between the duration of CD and the choroidal thicknesses at all measurement points. We think this result is related to age rather than the duration of the disease. Subfoveal choroidal thickness has been shown to be negatively correlated with age in various studies [24].

The high choroidal thickness levels of the patients following a gluten-free diet can be explained with decreased levels of disease activity, poor adherence to gluten-free diet, or the disease recovery process. Wahab et al. reported that intestine mucosal recovery after adopting a gluten-free diet occurred in 65% of patients within two years, in 85% within five years, and in 90% after five years following the diagnosis [25]. Gluten induces structural and inflammatory changes in most patients as quickly as 14 days after exposure [26].

PPE is a new clinical entity that was defined in 2013 [27]. Characteristics of the disease are increased choroidal thickening, pathologically dilated veins in Haller's layer, thinning in Sattler's and choriocapillaris layers, and the variety of retinal pigment epithelium abnormalities at the macula with a lack of subretinal fluid and drusen. Dansingani et al. reported that the choroidal thicknesses of eyes with the pachychoroid phenotype are greater than 300 *μ*m [28]. Several clinical manifestations have been described in the pachychoroid spectrum, including uncomplicated pachychoroid, PPE, central serous chorioretinopathy (CSCR), pachychoroid neovasculopathy, and polypoidal choroidal vasculopathy.

PPE is a forme fruste of CSCR. Saito et al. reported a patient with PPE who was later diagnosed with CSCR in the same eye during follow-up [29]. In another report, CSCR was observed in the fellow eye of a patient who was followed-up for PPE [28]. We encountered five patients with UCP in one eye and PPE in the fellow eye in the coeliac group. Although we could not find any CSCR findings in the coeliac group, observing PPE and UCP findings in the same patient suggests they may develop CSCR during follow-up.

This is the first study showing increased choroidal thickness and PPE associated with CD. The study's primary limitation is that we could not evaluate the effect of disease activity on choroidal thickness. We believe the choroidal thickness of newly diagnosed coeliac patients may be thicker than the coeliac patients following a gluten-free diet due to the decreased severity of inflammation. Disease activity can be assessed by measuring antiendomysium antibodies and tTG. Comparing pre- and postdietary choroidal thickness measurements in newly diagnosed coeliac patients may allow researchers to evaluate the relationship between disease activity and choroidal thickness more thoroughly.

In conclusion, it is probable that systemic inflammation in coeliac patients causes the enlargement of choroidal vessels and increasing choroidal thickness. EDI-OCT can be used as a noninvasive method for evaluating disease activity. We believe PPE is a clinical entity that should be kept in mind concerning CSCR development during follow-up with coeliac patients. Further investigations should be conducted to explain the relationship between the activity of the disease and choroidal thickness.

## Figures and Tables

**Table 1:** Demographic and ocular characteristics of coeliac and control groups.

Parameters	Coeliac group (*n*=70)	Control group (*n*=70)	*p* Value
Age (years)	37.4 ± 12.8	38.9 ± 11.2	0.601
Mean duration of disease (years)	4.6 ± 5.01	—	—
AL (mm)	22.6 ± 0.3	22.7 ± 0.3	0.314
IOP (mmHg)	15.7 ± 2.3	15.4 ± 2.2	0.822
Gender			1.0
Female	52	52	
Male	18	18	

**Table 2:** The mean ± SD choroidal thickness measurements.

Choroidal thickness locations	Coeliac group	Control group	*p* Value
Subfoveal	447.5 ± 85.9	282.5 ± 50.6	<0.001
Nasal 500 *µ*m	427.9 ± 88.0	269.1 ± 51.4	<0.001
Nasal 1000 *µ*m	404.4 ± 94.4	251.8 ± 52.2	<0.001
Nasal 1500 *µ*m	376.8 ± 97.4	229.2 ± 50.2	<0.001
Temporal 500 *µ*m	430.2 ± 82.4	276.8 ± 48.9	<0.001
Temporal 1000 *µ*m	409.8 ± 78.7	266.5 ± 49.6	<0.001
Temporal 1500 *µ*m	388.4 ± 80.6	253.6 ± 49.6	<0.001

**Table 3:** Correlation coefficient results in the coeliac group.

Choroidal thickness locations	Disease duration	Age	Gender
Subfoveal	*r* = 0.439, *p* = 0.008	*r*=−0.233, *p*=0.179	*r*=−0.182, *p*=0.297
Nasal 500 *µ*m	*r* = 0.417, *p* = 0.013	*r*=−0.232, *p*=0.179	*r*=−0.176, *p*=0.312
Nasal 1000 *µ*m	*r* = 0.418, *p* = 0.013	*r*=−0.207, *p*=0.232	*r*=−0.161, *p*=0.356
Nasal 1500 *µ*m	*r* = 0.430, *p* = 0.010	*r*=−0.175, *p*=0.314	*r*=−0.182, *p*=0.295
Temporal 1000 *µ*m	*r* = 0.393, *p* = 0.019	*r*=−0.334, *p*=0.050	*r*=−0.180, *p*=0.301
Temporal 1500 *µ*m	*r* = 0.388, *p* = 0.021	*r* = −0.343, *p* = 0.043	*r*=−0.225, *p*=0.193

## Data Availability

The data used to support the findings of this study are available from the corresponding author upon request.

## Additional Points

*Brief Summary Statement*. This article focusses on the choroidal thickness in CD. Thus, we aimed at evaluating choroidal thickness changes as a diagnostic and follow-up criterion in CD.

## References

[1] Troncone R., Discepolo V. (2014). Celiac disease and autoimmunity. *Journal of Pediatric Gastroenterology and Nutrition*.

[2] Gabrieli D., Ciccone F., Capannolo A. (2017). Subtypes of chronic gastritis in patients with celiac disease before and after gluten-free diet. *United European Gastroenterology Journal*.

[3] Sollid L. M. (2002). Coeliac disease: dissecting a complex inflammatory disorder. *Nature Reviews Immunology*.

[4] Sollid L. M., Qiao S.-W., Anderson R. P., Gianfrani C., Koning F. (2012). Nomenclature and listing of celiac disease relevant gluten T-cell epitopes restricted by HLA-DQ molecules. *Immunogenetics*.

[5] Cukrowska B., Sowińska A., Bierła J. B., Czarnowska E., Rybak A., Grzybowska-Chlebowczyk U. (2017). Intestinal epithelium, intraepithelial lymphocytes and the gut microbiota—key players in the pathogenesis of celiac disease. *World Journal of Gastroenterology*.

[6] van Bergen J., Mulder C. J., Mearin M. L., Koning F. (2015). Local communication among mucosal immune cells in patients with celiac disease. *Gastroenterology*.

[7] Uzel M. M., Citirik M., Kekilli M., Cicek P. (2017). Local ocular surface parameters in patients with systemic celiac disease. *Eye*.

[8] Erbasan F., Çoban D. T., Karasu U. (2017). Primary Sjögren’s syndrome in patients with celiac disease. *Turkish Journal of Medical Sciences*.

[9] Guandalini S., Schmitz J., Shmerling D. H., Visakorpi J. K. (1990). Revised criteria for diagnosis of coeliac disease: report of working group of European Society of Pediatric Gastroenterology and Nutrition. *Archives of Disease in Childhood*.

[10] Hosoda Y. I., Uji A., Hangai M., Morooka S., Nishijima K., Yoshimura N. (2014). Relationship between retinal lesions and inward choroidal bulging in Vogt-Koyanagi-Harada disease. *American Journal of Ophthalmology*.

[11] Gemenetzi M., De Salvo G., Lotery A. J. (2010). Central serous chorioretinopathy: an update on pathogenesis and treatment. *Eye*.

[12] Kneepkens C. M., von Blomberg B. M. (2012). Clinical practice: coeliac disease. *European Journal of Pediatrics*.

[13] Fina D., Sarra M., Caruso R. (2008). Interleukin 21 contributes to the mucosal T helper cell type 1 response in coeliac disease. *Gut*.

[14] Pietz G., De R., Hedberg M. (2017). Immunopathology of childhood celiac disease—key role of intestinal epithelial cells. *PLoS One*.

[15] Lahat N., Shapiro S., Karban A. (1999). Cytokine profile in coeliac disease. *Scandinavian Journal of Immunology*.

[16] Mollazadegan K., Kugelberg M., Tallstedt L., Ludvigsson J. F. (2012). Increased risk of uveitis in coeliac disease: a nationwide cohort study. *British Journal of Ophthalmology*.

[17] Keller J., Torres-Torres R., Sainz de la Maza M. (2013). Anterior scleritis and celiac disease: a proposed association. *Ocular Immunology and Inflammation*.

[18] Jampol L. M. (2008). Celiac disease presenting as a xerophthalmic fundus. *Retina*.

[19] Maruko I., Iida T., Sugano Y. (2011). Subfoveal choroidal thickness after treatment of Vogt-Koyanagi-Harada disease. *Retina*.

[20] Ishikawa S., Taguchi M., Muraoka T., Sakurai Y., Kanda T., Takeuchi M. (2014). Changes in subfoveal choroidal thickness associated with uveitis activity in patients with Behcet’s disease. *British Journal of Ophthalmology*.

[21] Gundogan F. C., Akay F., Uzun S., Ozge G., Toyran S., Gen H. (2016). Choroidal thickness changes in the acute attack period in patients with familial Mediterranean fever. *Ophthalmologica*.

[22] Ersan I., Kilic S., Arikan S. (2016). Evaluation of macular ganglion cell-inner plexiform layer and choroid in psoriasis patients using enhanced depth imaging spectral domain optical coherence tomography. *Ocular Immunology and Inflammation*.

[23] Tan C. S., Ouyang Y., Ruiz H., Sadda S. R. (2012). Diurnal variation of choroidal thickness in normal, healthy subjects measured by spectral domain optical coherence tomography. *Investigative Opthalmology & Visual Science*.

[24] Goldenberg D., Moisseiev E., Goldstein M., Loewenstein A., Barak A. (2012). Enhanced depth imaging optical coherence tomography: choroidal thickness and correlations with age, refractive error, and axial length. *Ophthalmic Surgery, Lasers, and Imaging*.

[25] Wahab P. J., Meijer J. W., Mulder C. J. (2002). Histologic follow-up of people with celiac disease on a gluten-free diet: slow and incomplete recovery. *American Journal of Clinical Pathology*.

[26] Leffler D., Schuppan D., Pallav K. (2013). Kinetics of the histological, serological and symptomatic responses to gluten challenge in adults with coeliac disease. *Gut*.

[27] Warrow D. J., Hoang Q. V., Freund K. B. (2013). Pachychoroid pigment epitheliopathy. *Retina*.

[28] Dansingani K. K., Balaratnasingam C., Naysan J., Freund K. B. (2016). En face imaging of pachychoroid spectrum disorders with swept-source optical coherence tomography. *Retina*.

[29] Saito W., Hashimoto Y., Hirooka K., Ishida S. (2018). Choroidal thickness changes in a patient diagnosed with central serous chorioretinopathy during follow-up for pachychoroid pigment epitheliopathy. *Retinal Cases & Brief Reports*.

